# Supplementary material for: Evaluation of four commercial tests for detecting ceftiofur in waste milk bulk tank samples
Source: PLoS One. 2019 Nov 12;14(11):e0224884. doi: 10.1371/journal.pone.0224884 (PMC6850555; doi:10.1371/journal.pone.0224884)
Supplement: S1 Table — (DOCX) [file pone.0224884.s001.docx]

**S1 Table**. LC-MS/MS limit of quantification for drug residues in milk.

|  | Limit of Quantification (ng/ml) |
| --- | --- |
| 5-hydroxyflunixin | 2 |
| Ampicillin | 10 |
| Bacitracin | 250 |
| Cephapirin | 10 |
| Ceftiofur | 10 |
| Chlortetracycline | 10 |
| Ciprofloxacin | 5 |
| Cloxacillin | 10 |
| Doxycycline | 10 |
| Enrofloxacin | 5 |
| Erythromycin | 50 |
| Oxytetracycline | 10 |
| Penicillin G | 5 |
| Sarafloxacin | 5 |
| Sulfachloropyridazine | 2 |
| Sulfadiazine | 2 |
| Sulfadimethoxine | 2 |
| Sulfamerazine | 2 |
| Sulfamethazine | 2 |
| Sulfapyridine | 2 |
| Sulfaquinoxaline | 2 |
| Sulfathiazole | 2 |
| Tetracycline | 10 |
| Thiabendazole | 10 |
| Tilmicosin | 10 |
| Tylosin | 10 |
| Virginiamycin | 5 |
