# Supplementary material for: Evaluation of four commercial tests for detecting ceftiofur in waste milk bulk tank samples
Source: PLoS One. 2019 Nov 12;14(11):e0224884. doi: 10.1371/journal.pone.0224884 (PMC6850555; doi:10.1371/journal.pone.0224884)
Supplement: S2 Table — For all calculation a significant level of α = 0.05 was used. (DOCX) [file pone.0224884.s002.docx]

**S2 Table.** Sample size output for quality parameters for waste milk samples (n=9). For all calculation a significant level of α = 0.05 was used.

| Parameters | SD^1^ | HD^2^ | Power^3^ |
| --- | --- | --- | --- |
| Fat (%) | 0.3 | 0.8 | 92 |
| Protein (%) | 0.06 | 0.4 | 99 |
| Lactose (%) | 0.05 | 0.15 | 96 |
| Solids-non-fat (%) | 0.04 | 0.2 | 99 |
| Somatic cell count (x10^3^ cell/mL) | 221 | 1000 | 99 |
| Coliform Count (CFU/ml) | 90 | 300 | 98 |
| Standard Plate count (x 10^3^ cfu/mL) | 12.6 | 30 | 86 |

1. Standard error.
2. Hypnotized difference to detect.
3. Percent probability of detecting a significant difference when one exists.
