# Supplementary material for: Evaluation of four commercial tests for detecting ceftiofur in waste milk bulk tank samples
Source: PLoS One. 2019 Nov 12;14(11):e0224884. doi: 10.1371/journal.pone.0224884 (PMC6850555; doi:10.1371/journal.pone.0224884)
Supplement: S3 Table — (DOCX) [file pone.0224884.s003.docx]

**S3 Table.** Quality parameters for waste milk samples (n=9).

| **Parameters** | **Mean (95% CI)*, Waste Milk** | **Mean, Whole Milk**** |
| --- | --- | --- |
| Fat (%) | 4.7 (3.4-5.9) | 4 |
| Protein (%) | 3.7 (3.4-3.9) | 3.1 |
| Lactose (%) | 4.3 (4.1-4.5) | 5 |
| Solids-non-fat (%) | 8.7 (8.5-8.9) | 8.9 |
| Somatic cell count (x10^3^ cell/mL) | 2,377.7 (1,457.0-3,298.5) |  |
| Coliform Count (CFU/ml) | 293 (81- 667) |  |
| Standard Plate count (x 10^3^ cfu/mL) | 92.0 (39.3-144.6) |  |

*95% confidence interval.

**Data from Godden, Sandra. "Colostrum management for dairy calves."*Veterinary Clinics of North America: Food Animal Practice* 24.1 (2008): 19-39.
