# Supplementary material for: Evaluation of four commercial tests for detecting ceftiofur in waste milk bulk tank samples
Source: PLoS One. 2019 Nov 12;14(11):e0224884. doi: 10.1371/journal.pone.0224884 (PMC6850555; doi:10.1371/journal.pone.0224884)
Supplement: S4 Table — Only variables for which a significant difference was observed was included in the table. (DOCX) [file pone.0224884.s004.docx]

**S4 Table**. Results from Kruskal-Wallis nonparametric test evaluating a significant difference between FP and TN results for each variable. Only variables for which a significant difference was observed was included in the table.

|  | **FP** | | **TN** | | ***P*-value**^3^ |
| --- | --- | --- | --- | --- | --- |
| **Test/variables** | **Score Sum**^1^ | **Score Mean**^2^ | **Score Sum** ^1^ | **Score Mean**^2^ |  |
| **SNAP^4^** |  |  |  |  |  |
| Lactose (%) | 227 | 11.9 | 151 | 18.9 | 0.037 |
| SCC (Cells/ml)* | 323 | 17 | 55 | 6.8 | 0.002 |
|  |  |  |  |  |  |
| **Delvo^5^** |  |  |  |  |  |
| Fat (%) | 151 | 10.1 | 226 | 18.8 | 0.004 |
| Protein (%) | 165 | 11 | 213 | 17.7 | 0.027 |
| SNF (%)** | 147 | 9.8 | 231 | 19.2 | 0.002 |
| Coliforms (CFU/ml) | 259 | 17.3 | 118 | 9.8 | 0.014 |
|  |  |  |  |  |  |
| **Penzyme^6^** |  |  |  |  |  |
| SPC (CFU/ml)*** | 104 | 9.5 | 273 | 17 | 0.013 |
|  |  |  |  |  |  |
| **Betastar^7^** |  |  |  |  |  |
| SCC (Cells/ml)* | 213 | 17.7 | 165 | 11.0 | 0.027 |
| Coliforms (CFU/ml) | 213 | 17.7 | 165 | 11.0 | 0.026 |

1. Score sum for each variable found to be significantly different between FP and TN results.

2. Score mean for each variable found to be significantly different between FP and TN results.

3. *P*-value for Kruskal-Wallis nonparametric test evaluating a significant difference between FP and TN results for each variable.

4. Penzyme® Milk Test

5. Betastar® Plus

6. Delvo® - SP

7. SNAP® β–lactam

* Somatic cell count

** Solids-non-fat

*** Standard plate count
